# The effects of Selenohomolanthionine supplementation on the rumen eukaryotic diversity of Shaanbei white cashmere wether goats

**DOI:** 10.1038/s41598-023-39953-2

**Published:** 2023-08-12

**Authors:** Longping Li, Lei Qu, Tuo Li

**Affiliations:** 1 Shaanxi Provincial Engineering and Technology Research Center of Cashmere Goats, Yulin University, Yulin, 719000 China; 2 College of Life Sciences, Yulin University, Yulin, 719000 China

**Keywords:** Fungi, Microbial communities, Metagenomics, Microbiome

## Abstract

Selenium (Se) is an important microelement for animal health. However, the knowledge about the effects of Se supplementation on rumen eukaryotic community remains less explored. In this study, the ruminal eukaryotic diversity in three months old Shaanbei white cashmere wether goats, with body weight (26.18 ± 2.71) kg, fed a basal diet [0.016 mg/kg Se dry matter (DM), control group (CG)] were compared to those animals given basal diet supplemented with different levels of organic Se in the form of Selenohomolanthionine (SeHLan), namely low Se group (LSE, 0.3 mg/kg DM), medium Se group (MSE, 0.6 mg/kg Se DM) and high Se group (HSE, 1.2 mg/kg DM) using 18S rRNA amplicon sequencing. Illumina sequencing generated 2,623,541 reads corresponding to 3123 operational taxonomic units (OTUs). Taxonomic analysis revealed that Eukaryota (77.95%) and Fungi (14.10%) were the dominant eukaryotic kingdom in all samples. The predominant rumen eukaryotic phylum was found to be Ciliophora (92.14%), while fungal phyla were dominated by Ascomycota (40.77%), Basidiomycota (23.77%), Mucoromycota (18.32%) and unidentified_Fungi (13.89%). The dominant eukaryotic genera were found to be *Entodinium* (55.44%), *Ophryoscolex* (10.51%) and *Polyplastron* (10.19%), while the fungal genera were dominanted by *Mucor* (15.39%), *Pichia* (9.88%), *Aspergillu* (8.24%), *Malassezia* (7.73%) and *unidentified_Neocallimastigaceae* (7.72%). The relative abundance of eukaryotic genera *Ophryoscolex*, *Enoploplastron* and fungal genus *Mucor* were found to differ significantly among the four treatment groups (*P* < 0.05). Moreover, Spearman correlation analysis revealed that the ciliate protozoa and fungi were negatively correlated with each other. The results of this study provided newer information about the effects of Se on rumen eukaryotic diversity patterns using 18s rRNA high-throughput sequencing technology.

## Introduction

Ruminants have an extremely diverse microbiota consisting of protozoa, fungi, archaea, bacteria and bacteriophage^1^. These rumen microorganisms act synergistically to help ruminants to efficiently degrade complex plant biomass such as cellulose, hemicellulose, lignin and pectin into absorbable compounds such as proteins, simple sugars and volatile fatty acids (VFAs), ultimately producing meat and milk for human needs^2^. Diets and additives directly affect rumen microbial population^3^. Numerous studies confirmed that trace elements affect gut health by modulating microbial community structure^4–6^. Essential trace element selenium (Se) as a component of glutathione peroxidase (GSH-Px) can protect cell membranes from peroxide damage^7^. Dietary Se supplementation has been confirmed to improve the antioxidant status of rumen microorganisms, thereby promoting rumen microbial growth and rumen fermentation, improve the apparent digestibility of nutrients, and ultimately promote the animal growth and production^8–11^. According to a report, sodium selenate-supplemented diet not only increased the abundance of amylolytic bacteria, sunch as *Ruminococcus* and *Fibrobacter*, but also promoted the activity of cellobiase, carboxymethyl cellulase, xylanase and protease^12^. Despite numerous studies have been conducted to understand the effect of dietary Se addition on the structure and diversity of rumen bacterial flora, there is a distinct lack of information about the effects of Se supplementation in diets on rumen eukaryotes. Mihaliková et al.^13^ demonstrated that supplementation of Se (disodium selenite or selenized yeast) in diet induced significant growth of the ruminal *Ophryoscolex* and *Diploplastron* population in sheep based on morphological observation and counting. To the best of our knowledge, limited study has focused on identifying and understanding the diversity of ruminal eukaryotes of receiving Se supplementation in ruminants using high-throughput sequencing technology, and this requires further research and investigation.

Protozoa and fungi are the main eukaryotic organisms in the rumen, account for up to 50% and 20% of the total rumen microbial biomass, respectively^14,15^. Rumen ciliated protozoa contribute to intra-ruminal circulation of microbial proteins^16^ and to degrade complex carbohydrate and produce volatile fatty acids, lactic acid, carbon dioxide and hydrogen enhancing methanogenesis in the rumen^17^. As a unicellular organisms, ciliated protozoa utilize the products from phagocytosis of bacteria to meet their nutritional needs^18^. Furthermore, ruminal protozoa (Litostomatea) is widely recognized for the role of cellulose degradation in the rumen^19^. Ruminal anaerobic fungi are capable of producing highly active fibrolytic enzymes that disrupt plant cell walls. A previous study showed that anaerobic fungi are widespread in the digestive tract of herbivores^20^. Fungal zoospores preferentially colonize lignin-rich regions of plant cell walls and dissolve these regions. These degradation products serve as substrates for fiber-degrading rumen bacteria and play an important role in further feed digestion and utilization^21^. However, due to its unique anatomical features and important functions of the rumen, in-depth studies of ruminant eukaryotic populations in ruminants are very scarce.

Previous studies on the effects of dietary Se supplementation for ruminants mostly focused on its deficiency, rumen bacterial community and animal growth. However, few studies reported the effects of dietary Se supplementation on the structure of ruminant eukaryotes. For the study of morphologically indistinguishable eukaryotic species, the previous studies showed that the eukaryotic 18S rRNA gene can be used as a molecular marker to identify different eukaryotes^22^. Our previous study has confirmed that dietary addition of Selenohomolanthionine (SeHLan) changed the rumen bacterial community structure and exerted a positive effect on the rumen bacterial metabolic function of Shaanbei white cashmere wether goats using 16s rRNA gene sequencing^11^. In the present study, we further evaluated the effects of dietary SeHLan supplementation on eukaryotic diversity of Shaanbei white cashmere wether goats using 18S rRNA amplicon sequencing. The results of our study will broaden our understanding and provide newer information of the effect of Se on rumen eukaryotic diversity in Shaanbei white cashmere goats.

## Results

### Rumen eukaryotic diversity

**Figure 1:**
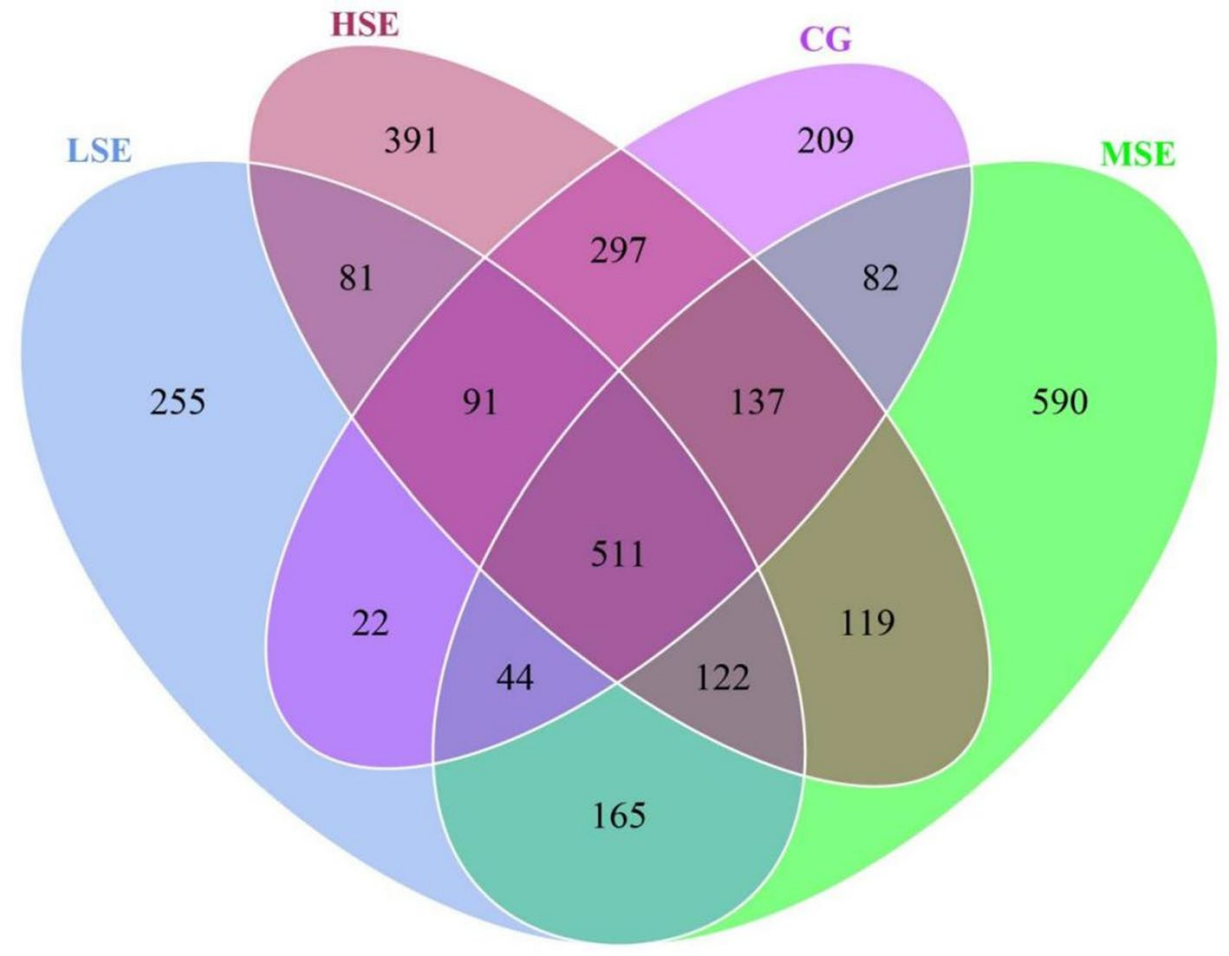
The Venn diagram of the shared and unique OTUs throughout the four different SeHLan added groups. CG, control group; LSE, low Se group; MSE, medium Se group; HSE, high Se group.

**Figure 2:**
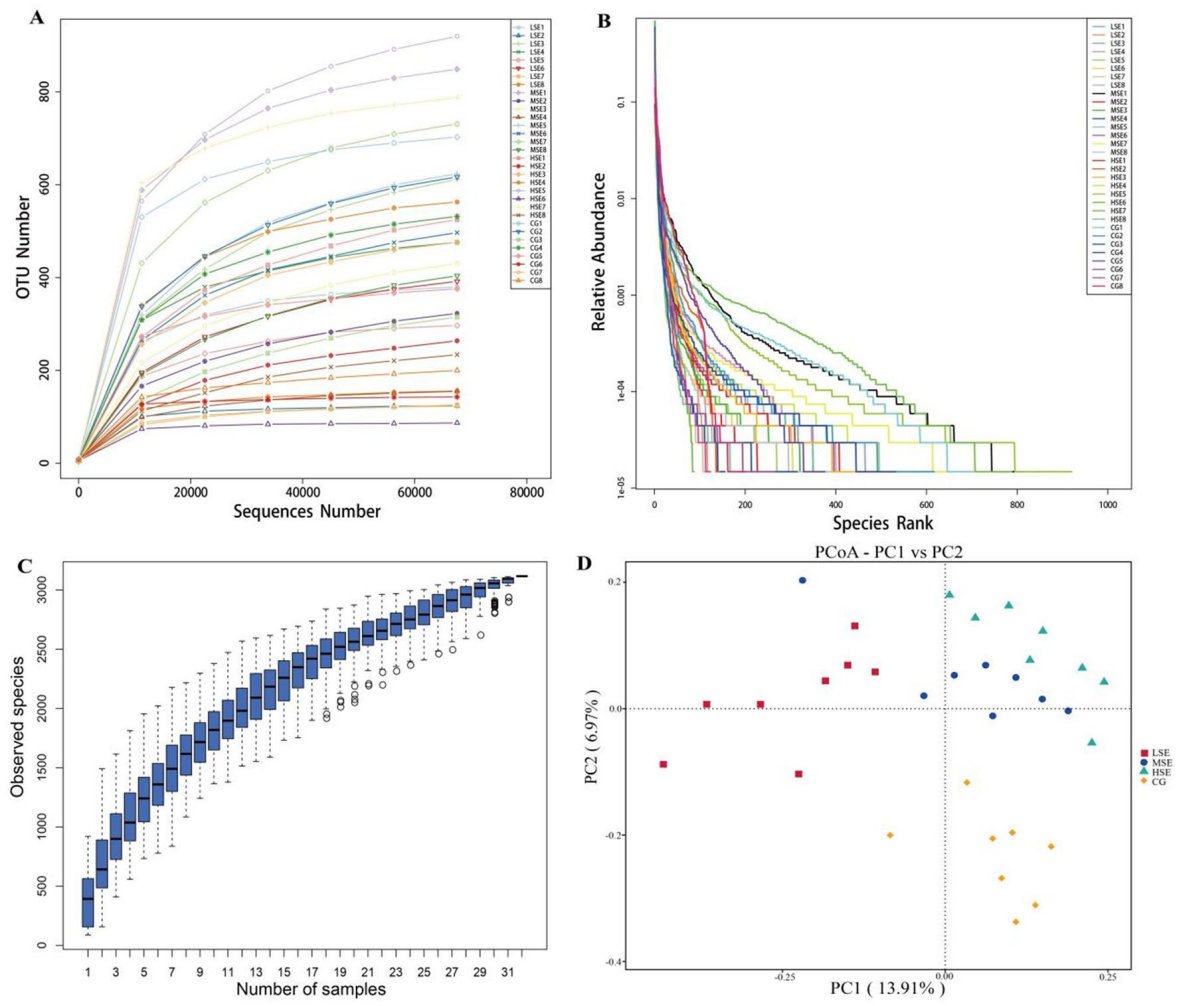
The alpha- and beta-diversity among four SeHLan supplemented groups. (**A**) Rarefaction curves based on V4 of 18S rRNA gene; (**B**) the rank abundance curves; (**C**) species accumulation curves; (**D**) principal coordinate analysis (PCoA) plot based on Bray–Curtis distance among four SeHLan supplemented groups. CG, control group; LSE, low Se group; MSE, medium Se group; HSE, high Se group.

The 18s rRNA gene sequencing experiment of 32 Shaanbei white cashmere wether goat rumen fluid samples produced a total of 2,623,541 reads with an average of 81,985.66 ± 765.86 [(mean ± standard error of the mean (SEM), n = 32] per sample. A total of 3,123 operational taxonomic units (OTUs) were obtained based on the 97% similarity threshold in the present study. The number of unique OTU in CG, LSE, MSE and HSE groups were 209 (6.69%), 255 (8.17%), 590 (18.89%), 391 (12.52%), respectively (Fig. 1). The MSE group had the highest number of OTUs, and the LSE group had the lowest number of OTUs. 511 OTUs (16.36% of the total) were shared among four different level SeHLan added groups. The rarefaction curves (Fig. 2A) and the rank abundance (Fig. 2B) indicated that our sequencing depth is sufficient to contain most of the microbial information and sampling quality met the requirements for sequencing and analysis. Additionally, the species accumulation curves showed that our samples were sufficient for OTUs test and could predict the species richness of samples (Fig. 2C). Various alpha-diversity estimators were compared among 4 treatments. We found that the values of observed species, Chao1, ACE and PD_whole_tree were all highest in the rumen population when goats received moderate doses of SeHLan (MSE group), but Shannon and Simpson index values were all increases when SeHLan supplementation, which point out that higher eukaryotic diversity and abundance with the increase of dietary SeHLan addition (Table 1). However, there was no significant difference were observed for the above-mentioned alpha-diversity indexes (*P* > 0.05). Furthermore, the Good’s coverage index value in LSE significantly higher than that in the MSE group (*P* < 0.05). The PcoA based on Bray–Curtis dissimilarity matrices showed a clear differences of the eukaryotic communities between control group and SeHLan supplemented groups (Fig. 2D).

**Table 1:** Operational taxonomic unit count and α diversity estimation based on the 18S rRNA gene sequencing analysis of the four different treatment groups.

Item	Treatments^1^				SEM	*P*-value
	CG	LSE	MSE	HSE		
Observed species	391.25 ± 73.04	320.38 ± 62.94	501.63 ± 79.62	467.00 ± 105.61	40.87	0.418
Shannon	4.44 ± 0.38	3.79 ± 0.25	3.96 ± 0.46	4.44 ± 0.48	0.19	0.564
Simpson	0.869 ± 0.038	0.813 ± 0.051	0.841 ± 0.028	0.866 ± 0.028	0.018	0.694
Chao1	433.92 ± 77.12	350.92 ± 73.02	566.13 ± 83.73	505.52 ± 112.07	44.16	0.359
ACE	443.35 ± 76.08	356.92 ± 74.52	571.73 ± 82.67	511.53 ± 113.07	44.20	0.365
Good’s coverage	0.9988 ± 0.0002^b^	0.9995 ± 0.00027 ^a^	0.9984 ± 0.00026^b^	0.9990 ± 0.00027^b^	0.00014	0.037
PD_whole_tree	92.62 ± 12.26	82.18 ± 14.97	108.39 ± 14.88	104.34 ± 18.10	7.45	0.614

Values in the same row with different lower-case letters mean significant difference among the treatments (*P* < 0.05), while same letters or no letters in the same row means not significant difference among treatments (*P* > 0.05).

^1^CG, LSE, MSE, and HSE: treatment groups supplemented with Se at 0 (control), 0.30 (low), 0.60 (medium), and 1.2 (high) mg/kg dry matter, respectively.

At the taxonomic level, a total of 6 kingdom, 55 phyla, 182 classes, 341 orders, 391 families, 691 genera and 737 species were identified in all samples from our data. Ruminal eukaryotes analyses revealed that 6 kingdoms were present in all samples. Out of those 6 kingdom, Eukaryota (CG 62.0%, LSE 87.4%, MSE 83.5%, and HSE 78.9%, respectively) and Fungi (CG 25.0%, LSE 9.0%, MSE 9.5%, and HSE 12.5%, respectively) were the most dominant eukaryotic kingdom in all samples and rumen eukaryotes are dominated by ciliated protozoa (belonging to kingdom of Eukaryota) and fungi. The results suggest that with the dietary SeHLan addition level increasing, the relative abundance of rumen Eukaryota gradually decreases, while the relative abundance of fungi gradually increases in general. The taxonomical composition of ruminal eukaryotes was investigated by sequencing the V4 region of the 18S rRNA gene on the rumen samples with the results uploaded to the NCBI’s sequence read archive (PRJNA846111).

### Composition and comparison of ruminal ciliated protozoa

**Figure 3:**
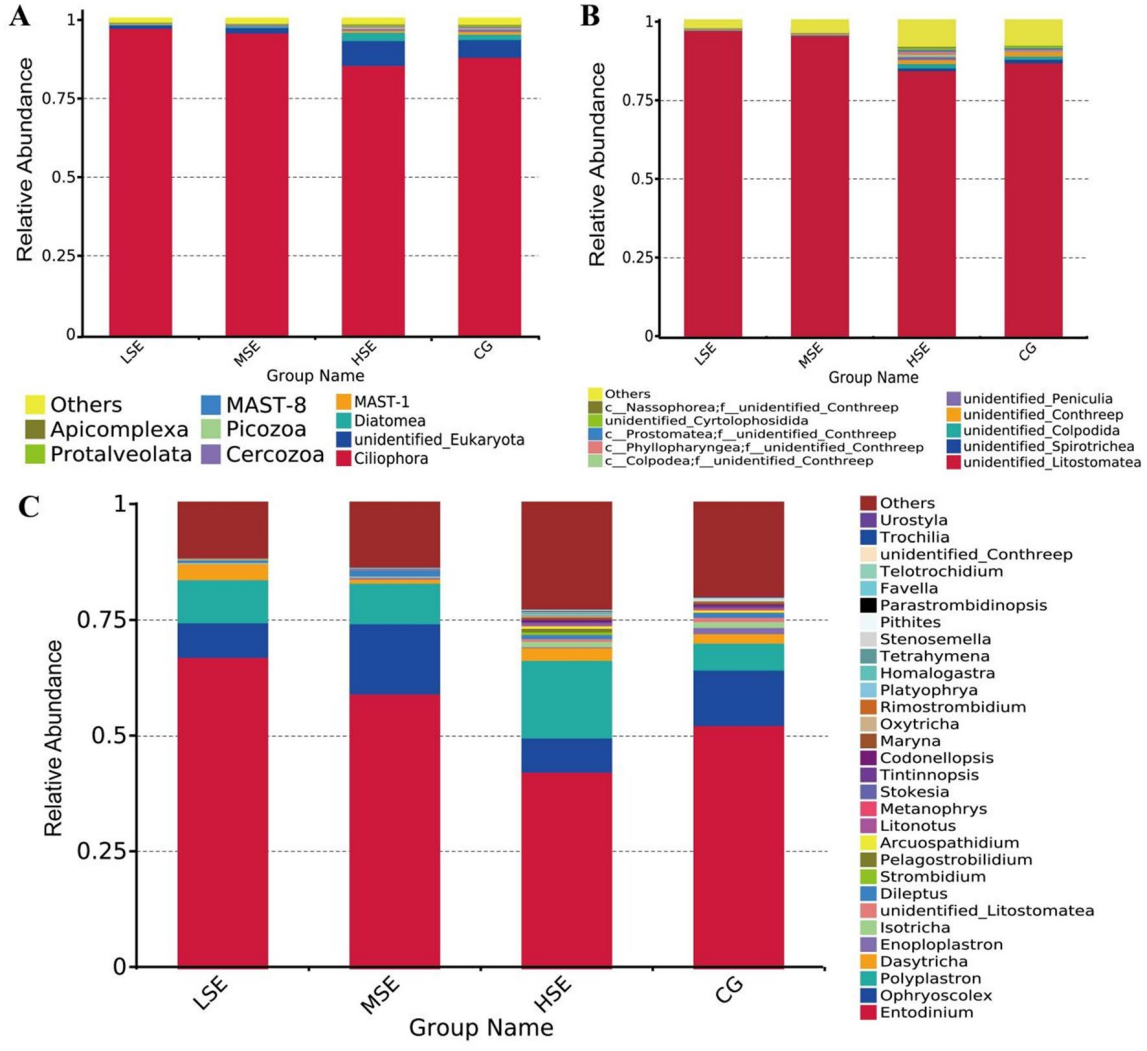
Rumen eukaryotic compositional profiles of different SeHLan supplemented groups. Average relative abundance of eukaryotic taxa at phylum level (**A**), family level (**B**), and genus level (**C**). CG, control group; LSE, low Se group; MSE, medium Se group; HSE, high Se group.

The dominant eukaryotic phylum in all four treatment groups was Ciliophora (CG 88.68%, LSE 97.91%, MSE 96.45%, and HSE 86.61%, respectively) (Fig. 3A, Table S1). At the family level, unidentified_Litostomatea (CG 87.47%, LSE 97.72%, MSE 96.07% and HSE 84.99%, respectively) was the most highly represented taxa (Fig. 3B, Table S2). At the genus level, among the detected 691 genera, 7 the most abundant genera (average relative abundance of > 1% for at least one group) were found in all groups. Three dominant genera (average relative abundance of > 5% for at least one group) were *Entodinium* (CG 60.09%, LSE 68.82%, MSE 61.63%, and HSE 49.78%, respectively), *Ophryoscolex* (CG 12.57%, LSE 7.62%, MSE 15.51%, and HSE 7.87%, respectively) and *Polyplastron* (CG 6.31%, LSE 9.48%, MSE 9.02%, and HSE 18.27%, respectively) (Fig. 3C, Table S3).

**Figure 4:**
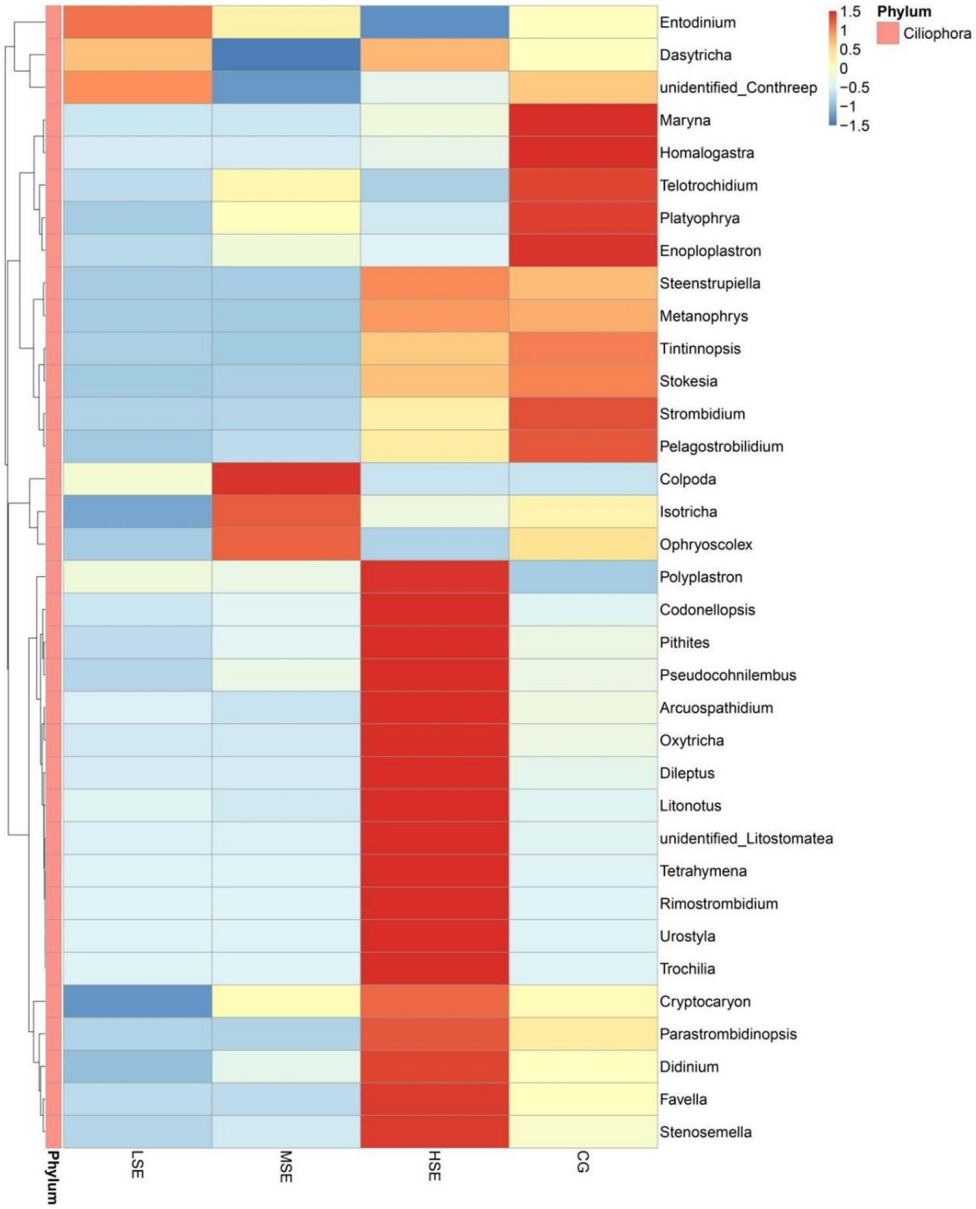
Heatmap analysis of top 35 eukaryotic genera as determined by the relative abundance of taxonomic genera levels. The vertical axis is sample information, the horizontal axis is species annotation information. On the left is the species clustering tree. The heatmap in the middle corresponds to the standardized Z-score of the relative abundance of each row species.

The differences in relative abundance of *Ophryoscolex* and *Enoploplastron* in four groups were statistically significant (*P* < 0.05) (Table S3). To provide clarity and visualization, we constructed a heatmap which based on the relative abundances of top 35 eukaryotic genera (Fig. 4). Based on heatmap, the results clearly showed that the relative abundances of *Maryna*, *Homalogastra*, *Telotrochidium, Platyophrya*, *Enoploplastron, Strombidium* and *Pelagostrobilidium* were all higher in the CG group than other three groups which were all SeHLan supplement. However, the relative abundance of *Entodinium*, *Dasytricha* and *unidentified_Conthreep* were correlated positively with the LSE group (low SeHLan addition); *Colpoda, Isotricha* and *Ophryoscolex* were correlated positively with the MSE group (medium SeHLan addition) and the HSE group (high SeHLan addition) correlated positively with the relative abundance of *Polyplastron, Codonellopsis, Pithites, Pseudocohnilembus, Arcuospathidium, Oxytricha, Dileptus, Litonotus, unidentified_Litostomatea, Tetrahymena, Rimostrombidium, Urostyla, Trochilia, Cryptocaryon, Parastrombidinopsis, Didinium, Favella,* and* Stenosemella.*

### Composition and comparison of ruminal fungi

**Figure 5:**
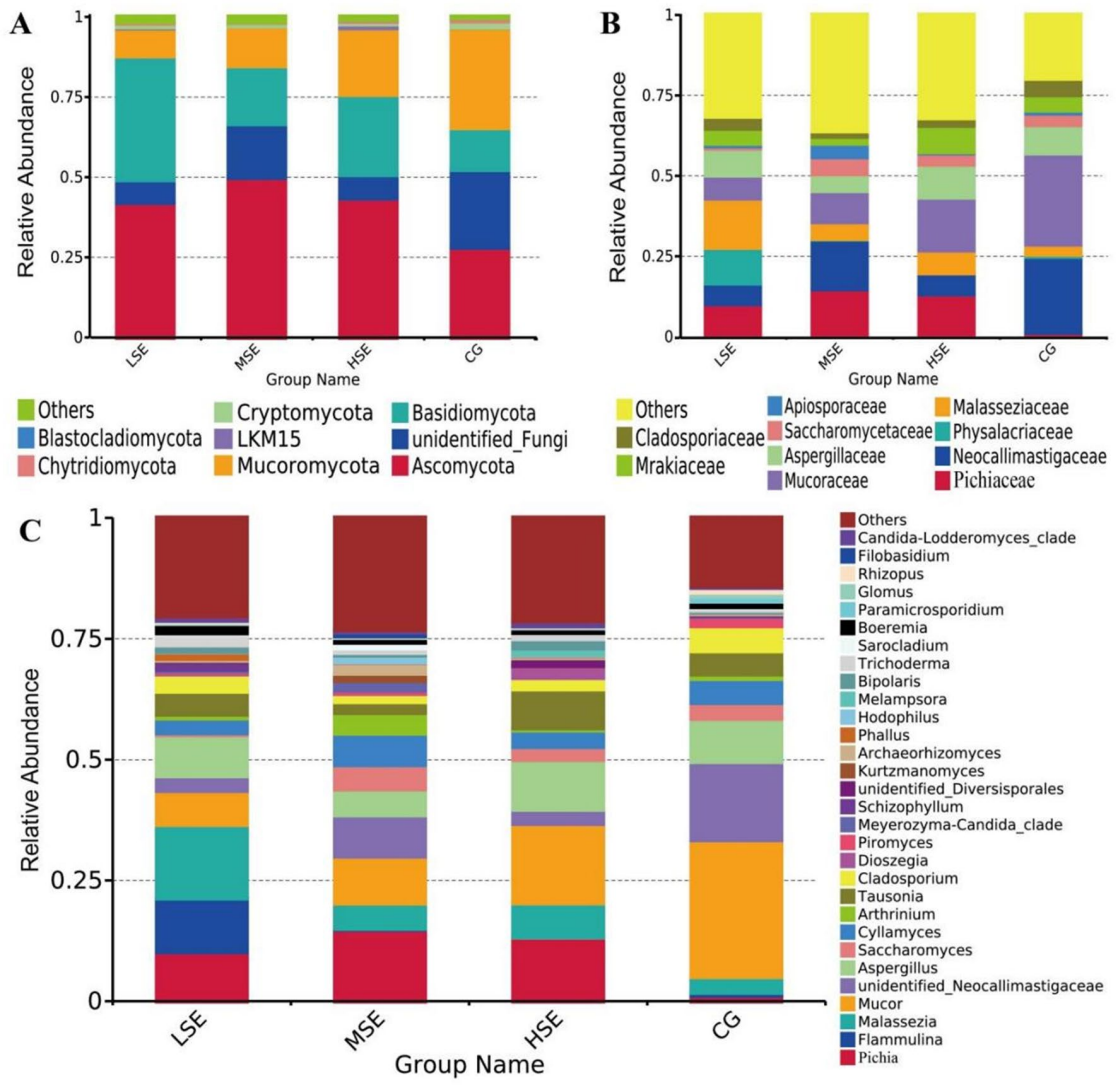
Rumen fungal compositional profiles of different SeHLan supplemented groups. Average relative abundance of fungal taxa at phylum level (**A**), family level (**B**), and genus level (**C**). CG, control group; LSE, low Se group; MSE, medium Se group; HSE, high Se group.

The dominant fungal phyla included Ascomycota (CG 27.89%, LSE 42.29%, MSE 49.55%, and HSE 43.33%, respectively), unidentified_Fungi (CG 24.26%, LSE 7.07%, MSE 16.85%, and HSE 7.39%, respectively), Basidiomycota (CG 13.39%, LSE 38.66%, MSE 18.18%, and HSE 24.83%, respectively) and Mucoromycota (CG 31.37%, LSE 8.55%, MSE 12.52%, and HSE 20.85%, respectively) (Fig. 5A, Table S1). At the family level, Neocallimastigaceae (CG 23.49%, LSE 6.42%, MSE 15.61%, and HSE 6.65%, respectively), Pichiaceae (CG 1.21%, LSE 10.23%, MSE 14.85%, and HSE 13.24%, respectively), Physalacriaceae (CG 0.62%, LSE 11.09%, MSE 0.19%, and HSE 0.008%, respectively), Malasseziaceae (CG 3.41%, LSE 15.27%, MSE 5.21%, and HSE 7.02%, respectively), Mucoraceae (CG 28.43%, LSE 7.09%, MSE 9.73%, and HSE 16.33%, respectively), and Aspergillaceae (CG 8.89%, LSE 8.49%, MSE 5.11%, and HSE 10.47%, respectively) were the dominant families; other families included Saccharomycetaceae (CG 3.58%, LSE 0.79%, MSE 5.19%, and HSE 3.33%, respectively), Apiosporaceae (CG 0.94%, LSE 0.77%, MSE 4.25%, and HSE 0.39%, respectively), Mrakiaceae (CG 4.89%, LSE 4.72%, MSE 2.19%, and HSE 8.13%, respectively), and Cladosporiaceae (CG 5.09%, LSE 3.75%, MSE 1.70%, and HSE 2.39%, respectively) (Fig. 5B, Table S2). At the genus level, the dominant fungal genera included *Pichia* (CG 1.21%, LSE 10.23%, MSE 14.85%, and HSE 13.24%, respectively), *Flammulina* (CG 0.62%, LSE 11.09%, MSE 0.19%, and HSE 0.008%, respectively), *Malassezia* (CG 3.41%, LSE 15.27%, MSE 5.21%, and HSE 7.02%, respectively), *Mucor* (CG 28.43%, LSE 7.07%, MSE 9.73%, and HSE 16.33%, respectively), *unidentified_Neocallimastigaceae* (CG 16.25%, LSE 3.06%, MSE 8.52%, and HSE 3.04%, respectively), and *Aspergillus* (CG 8.89%, LSE 8.49%, MSE 5.11%, and HSE 10.47%, respectively), followed by *Saccharomyces* (CG 3.41%, LSE 0.54%, MSE 5.01%, and HSE 2.62%, respectively), *Cyllamyces* (CG 4.96%, LSE 3.06%, MSE 6.52%, and HSE 3.41%, respectively), *Arthrinium* (CG 0.94%, LSE 0.77%, MSE 4.25%, and HSE 0.39%, respectively), and *Tausonia* (CG 4.89%, LSE 4.72%, MSE 2.19%, and HSE 8.08%, respectively) (Fig. 5C, Table S3).

**Figure 6:**
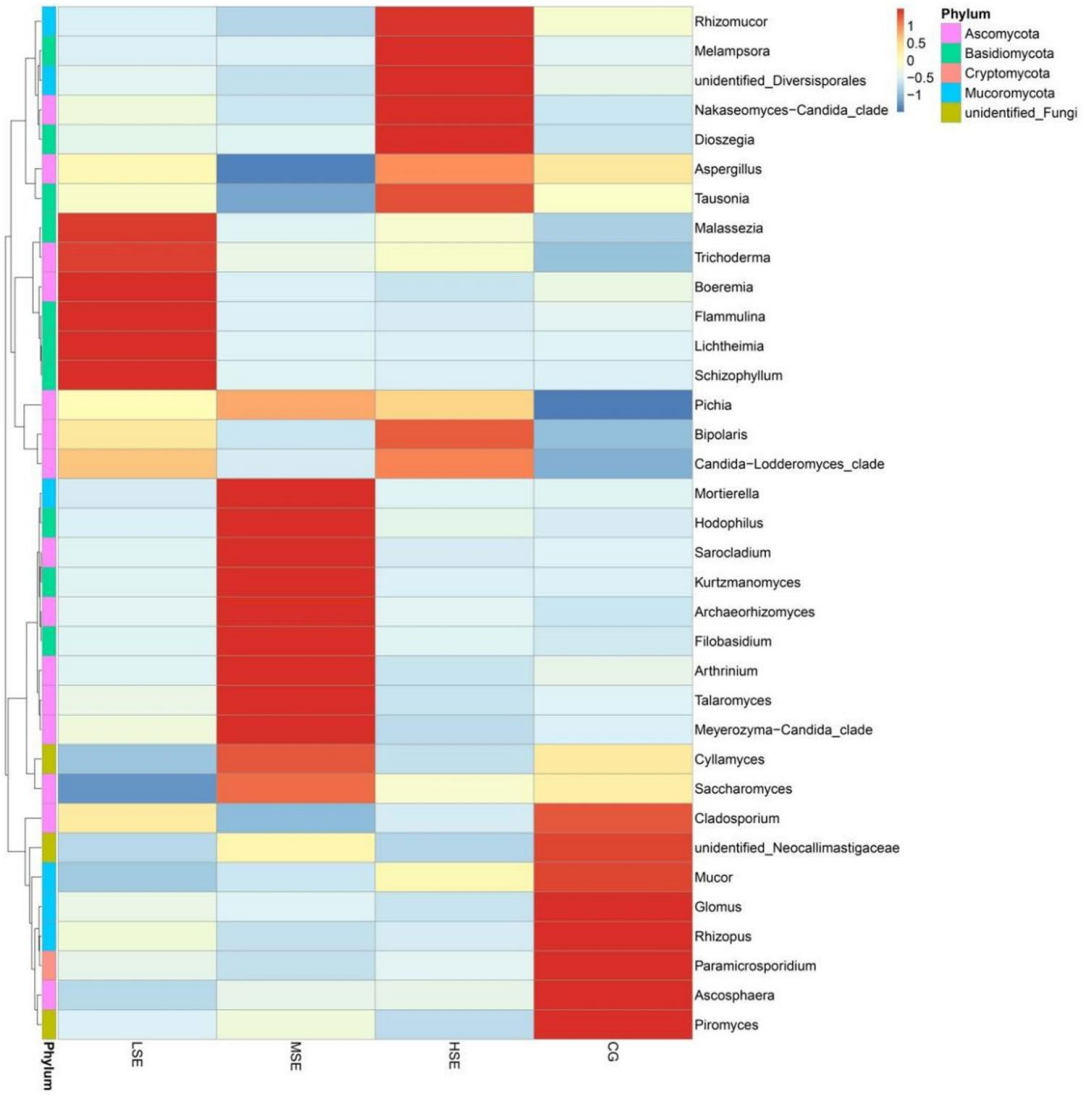
Heatmap analysis of top 35 fungal genera as determined by the relative abundance of taxonomic genera levels. The vertical axis is sample information, the horizontal axis is species annotation information. On the left is the species clustering tree. The heatmap in the middle corresponds to the standardized Z-score of the relative abundance of each row species.

The differences in relative abundance of family Mucoraceae and genus *Mucor* in differnent groups were statistically significant (*P* < 0.05) (Table S3). To provide clarity and visualization, we constructed a heatmap which based on the relative abundances of top 35 fungal genera (Fig. 6). Based on heatmap, the results clearly showed that the relative abundances of *Cladosporium*, *unidentified_Neocallimastigaceae*, *Mucor*, *Glomus*, *Rhizopus*, *Paramicrosporidium*, *Ascosphaera*, *Piromyces* were all higher in the CG group than other three groups. While the relative abundance of *Malassezia*, *Trichoderma*, *Boeremia*, *Flammulina*, *Lichtheimia* and *Schizophyllum* were correlated positively with the LSE group (low SeHLan addition); the abundance of *Mortierella*, *Hodophilus*, *Sarocladium*, *Kurtzmanomyces*, *Archaeorhizomyces*, *Filobasidium*, *Arthrinium*, *Talaromyce*s, and *Meyerozyma-Candida_clade* were correlated positively with the MSE group (medium SeHLan addition) and the HSE group (high SeHLan addition) correlated positively with the relative abundance of *Rhizomucor*, *Melampsora*, *unidentified_Diversisporales*, *Nakaseomyces-Candida_clade*, *Dioszegia, Tausonia* and *Bipolaris.*

### Species interaction network analysis

**Figure 7:**
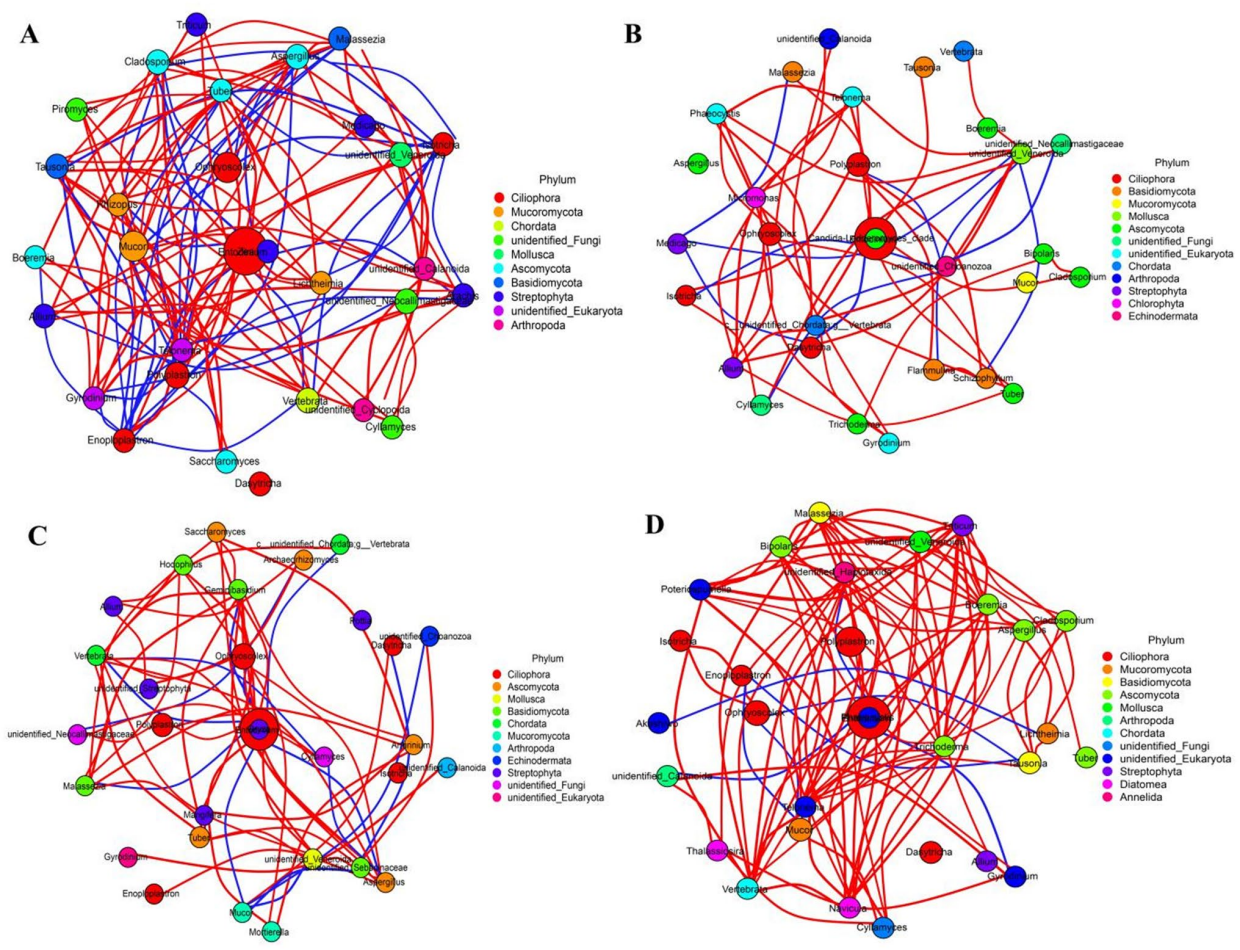
The rumen eukaryotes interaction network analysis among the control group (**A**), low Se group (**B**), medium Se group (**C**) and high Se group (**D**). Each circle represents a eukaryote, the size of the circle represents its relative abundance, different color represents different classification at phylum level, the line between the circles represents a significant correlation between the two eukaryotes (*P* < 0.05), the red line represents a positive correlation, while the blue line represents a negative correlation; the thicker of the line, the greater the value of the corresponding correlation coefficient.

Taxonomical data was amalgamated for each group and relative proportions of the eukaryotes at the genus level in each group were correlated by calculated the Spearman correlation coefficient method through R software (Version 2.15.3) with igraph package (Fig. 7). For CG group, genera like *Entodinium*, under phylum Ciliophora were negatively correlated with Fungi, such as *Cladosporium* (r: − 0.93, *P* = 8.63E−04), *Aspergillus* (r: − 0.83, *P* = 0.0102) and *Rhizopus* (r: − 0.88, *P* = 0.0041). *Cladosporium*, *Aspergillus* and *Malassezia* which all belong to fungi exhibited a high positive correlation with each other (r > 0.90, *P* < 0.0001) (Fig. 7A). In the LSE group, most phylum Ciliophora and fungi were exhibited positive correlations with each other, respectively, such as *Isotricha* was significantly associated with *Polyplastron* (r: 0.905, *P* = 0.002), and *Schizophyllum* was positively correlated with *Flammulina* (r: 0.966, *P* = 9.57E−05) (Fig. 7B). Most of fungal genera exhibited positive correlations with each other in the MSE group, specifically with *Geminibasidium* and *Malassezia* (r: 0.901, *P* = 0.0022), while genera *Entodinium* of Ciliophora demonstrated a negative correlation with most fungal genera like *Aspergillus* (r: − 0.83, *P* = 0.0101) and *Geminibasidium* (r: − 0.85, *P* = 0.0074) (Fig. 7C). Most genera of fungi or protozoa identified in the HSE group exhibited a high positive correlation within each other, like fungal genera *Boeremia* with *Malassezia* (r: 0.93, *P* = 7.16E−04), genera of ciliate protozoa *Enoploplastron* with *Polyplastron* (r: 0.85, *P* = 0.0069) and *Isotricha* (r: 0.93, *P* = 9.16E−04), respectively (Fig. 7D).

## Discussion

Previously, most studies on rumen eukaryotes, especially for protozoa, were morphologically observed with the aid of microscopes^23^. Studies on the rumen eukaryotic ecology and taxonomy have also been hampered by the difficulties in culture and their polymorphism. Additionally, scarce data is available on the effects of dietary selenium supplementation on rumen eukaryotic diversity compared to rumen bacteria or other natural environments. In recent years, due to the rapid development of molecular biology technology and high-throughput sequencing technology, 18S rRNA gene has been used as an important eukaryotic marker gene to detect uncultured species for better analysis and study of the diversity and evolutionary relationships of ruminal eukaryotes^24^. Among the nine interspersed hypervariable regions of 18S rRNA gene for eukaryotic diversity investigation, V4 and V9 regions are mainly used as molecular markers for the analysis of eukaryotic diversity^25^. In the present study, we conducted 70d feed experiment with different SeHLan supplementation (control, low, medium and high Se concentrations) in diets and the rumen eukaryotic diversity was analyzed using sequence the V4 hypervariable region of 18S rRNA gene. The obtained eukaryotic diversity and richness indices, including Observed species, Chao1 and ACE, PD_whole_tree, of MSE group were all higher than those of other three groups. However, the α-diversity indices (Observed species, Shannon, Simpson, Chao1, ACE, PD_whole_tree) were not significantly different among four treatment groups, indicating that SeHLan supplementation did not affect the diversity and abundance of rumen eukaryotic communities of Shaanbei white cashmere wether goats. Studies of growing puppies^26^, Tibetan Sheep^10^ and goats^11^ who reported that the alpha-diversity index of the gut and rumen bacterial community were also not affected by the selenite- or SeY-supplemented diets, those results are consistent with ours.

In the present study, the dominant abundance of eukaryotic phyla are Eukaryota of Ciliophora, followed by Fungi of Ascomycota, Basidiomycota, Mucoromycota and unidentified_Fungi, which accounted for more than 86% of the total eukaryotic populations. Amongst them, Ciliophora accounted for more than 75% of total eukaryotes. Several previous studies have reported that Ciliophora as the rumen dominant eukaryotes in Camel^22^, cattle^27^, goat and sheep^28^ and other ruminants^29^, those studies are consistent with our results. Further, overwhelming majority of the sequence variants of Ciliophora were assigned to genera *Entodinium*, *Ophryoscolex* and *Polyplastron* in the present study. This is consistent with the results reported by Shin et al.^30^ who found that *Entodinium* as the dominant protozoan genus in the rumen of cows. However, a study by Mishra et al.^22^ who reported that most of sequence of Ciliophora were taxonomically unassigned genera, and a small number of sequences were belonged to *Entodinium* in the rumen of camel. The reasons for those inconsistent results may be related to different experimental animal breeds or diet. Several early studies have confirmed that ruminal ciliate protozoa play an important role in promoting or protecting ruminal strictly anaerobic fermentation, especially for cellulolytic bacteria, by scavenging dissolved oxygen that enters the rumen together with the ingested feed, drinking water or saliva^31,32^. Ciliate protozoa are also responsible for acetogenesis and nitrogen fixation or methanogenesis^33^, defense against predators^34^ or as nutrients for host cells^35^. In addition, rumen ciliates also play an important role in maintaining prokaryotic population structure and diversity in the rumen and promoting efficient bacterial nitrogen use for the host by digesting and fermenting feed and shaping rumen microbiome^17^.

Fungi have a low rumen biomass and they are thought to be a minor contribution for plant degradation, although they also produce specific enzymes^14^. In this study, we found that with the increase of dietary SeHLan addition, the relative abundance of Ciliophora was decreased, the relative abundance of fungi of Ascomycota showed a trend of first increasing and then decreasing, while the relative abundance of fungi of Basidiomycota first decreased and eventually increased. However, the proportion of phyla, including Ciliophora, fungi of Ascomycota and Basidiomycota, in the SeHLan added groups (LSE, MSE, HSE) were all higher than that in the control group. The relative abundance of fungi of Mucoromycota increased with the SeHLan supplementation level, while the relative abundance of fungi of unidentified_Fungi showed a increasing to decreasing trend with an increase in dietary SeHLan supplementation. However, the proportion of phyla, including fungi of Mucoromycota and unidentified_Fungi, in the control group were all higher than that in the Se-supplemented diets (LSE, MSE, HSE). Those results indicated that the diet supplemented with a low or medium level of SeHLan may be more suitable for the survival of the phyla Ciliophora, fungi of Basidiomycota, and fungi of Ascomycota. However, SeHLan supplemented diets may inhibit the growth of rumen fungi of Mucoromycota and unidentified_Fungi. Previous research has also confirmed that rumen microbial population is capable of incorporating selenium into their cells and ruminal protozoa can influence animal selenium status^36^. Our previous study indicated that dietary supplementation of low (LSE) and medium (MSE) SeHLan could effectively increase the body weight, average daily gain, improve the nutrients utilization and food conversion ratio of Shaanbei white cashmere wether goats, while high SeHLan (HSE) is not beneficial for cashmere goat growth^11^. Therefore, we can speculate that addition of SeHLan to the diet changed the structure of the rumen eukaryotic population of Shaanbei white cashmere goats, thereby improving the feed utilization efficiency and promoting production, and Ciliophora, Ascomycota and Mucoromycota are mainly responsible for selenium utilization and have beneficial effects on the rumen eukaryotic organisms. However, this assumption requires further investigation.

Taxonomic classification in our study showed that the relative abundance of genus *Entodinium* was found gradually decreased with SeHLan supplemented level. The relative abundance of genus *Ophryoscolex* first increased and eventually decreased with dietary SeHLan addition, while the relative abundance of genus *Polyplastron* was found gradually increased with SeHLan-supplemented level. In general, compared with the control group, a higher proportion of genera *Entodinium*, *Ophryoscolex* and *Polyplastron* were found in the rumen of three SeHLan supplemented group’s goats. Our results are consistent with previous reports by Mihaliková et al.^13^ and Naziroglu et al.^37^ who also found that supplementation of feed with inorganic (sodium Se) or organic Se (yeast Se) induced significantly growth of genera *Entodinium*, *Polyplastron* and *Ophryoscolex* in the rumen of lambs. Previous studies also confirmed that Se has a stimulating effects on the growth of some rumen ciliate genera. Akbar et al.^38^ investigated that lactating Murrah buffaloes fed Se enriched diets had the significantly higher protozoan count. Fujihara et al.^36^ also reported that when a diet supplemented with 0.2 mg/kg Se in the form of selenite, rumen protozoa could capture 0.52 mg Se/kg of protozoan dry matter in sheep. As we all known, Se as a component of glutathione peroxidase (GSH-Px) can protect cell membranes from peroxide damage. Therefore, we can speculate that the explanation for higher abundance of *Entodinium*, *Ophryoscolex* and *Polyplastron* in the rumen of three SeHLan supplemented group’s goats may be due to the effects of Se on the antioxidant status of the rumen *Entodinium*, *Ophryoscolex* and *Polyplastron*. It means that the appropriate amount of SeHLan added to experimental goats might induced higher activity of selenoenzymes in the rumen protozoa. However, this assumption requires further investigation for the activity of rumen protozoal selenoenzymes.

According to a previous study, Kisidayova et al.^39^ reported that the growth of *O. caudatus f. tricoronatus* and *Entodunium caudatum* were inhibited at dietary Se supplementation level equal to and/or higher than 0.1 and 0.5 mg/L. Our results also suggest that dietary supplementation with high level of SeHLan decreased the *Entodinium* abundance, whereas supplementing the diets with low or medium level of SeHLan may be beneficial for promoting growth of genus *Entodinium,* which is the predominant rumen eukaryotic genus belong to Phylum Ciliophora in the present study. As the smallest, simplest and common rumen ciliates, *Entodinium* is present in the rumen of almost all ruminants, and the main function of *Entodinium* is to degrade starch which is essential for maintaining its growth^40^. The species belonging to the genus *Entodinium* was found engulfed starch and further fermented into acetic acid and butyric acid, which together account for 80–90% of total volatile fatty acids, while producing smaller amounts of formic acid, propionic acid, carbon dioxide and hydrogen^41^. In addition, *Entodinium* has a great potential to engulf and degrade bacteria^42^ contributing to the bacterial nitrogen turnover^17^ and lactic acid metabolism^40^. A previous study confirmed that the abundance of genus *Entodinium* was higher in low-RFI (residual feed intake) steers than that in high-RFI steers^43^. Yuste et al.^44^ reported that the genus *Entodinium* account for 93% of the total rumen protozoal community when beef cattle fed a high concentrate based diet. Therefore, high abundance of genus *Entodinium* in the rumen may play an important role in regulating rumen lactic acid metabolism, stabilizing rumen fermentation and improving animal feed utilization efficiency. In the present study, the relative abundance of *Entodinium* decreased with the dietary SeHLan-supplemented level, and low or medium level dietary Se supplementation stimulates the growth of *Entodinium* in the rumen of Shaanbei white cashmere wether goats. The *Ophryoscolex* and *Polyplastron* population were all higher in the SeHLan-supplemented groups than control group which is also supposed to dietary Se addition improved the antioxidant properties and ultimately promoted ruminal protozoa growth^13,45^.

Se affects rumen bacterial abundance and ultimately affects rumen microbial fermentation, while few studies have addressed the effects of Se on rumen eukaryotic populations or the composition and function of the eukaryotic population (protozoa, fungi) associated with rumen prokaryotic community (bacteria, archaea) and the extent of this association. In this study, we found that the major ciliate protozoa and fungi were negatively correlated with each other, suggesting that Se affects the interrelationship between rumen protozoa and fungi. However, this assumption needs further more investigation. Previous research confirmed that protozoa associated prokaryotes displayed a variety of functions, such as the adaptability of host cells^34,46^, nitrogen fixation^47^, methanogenesis^48^, acetogenesis^33^, provide a defense against predators^49^, and provide nutrients to host cells^35,46^. Therefore, other scientific methods such as metagenomics and metatranscriptomics analysis are required to further characterization of the prokaryotic communities associated with the eukaryotic population and their composition and roles in the rumen receiving Se supplemented in the diet.

## Conclusion

The results of this study confirmed that Se has a stimulating effects on the growth of some rumen ciliate and fungal genera. Dietary supplementation SeHLan increased the relative abundance of Eukaryota, while decreased the relative abundance of Fungi. The relative abundance of protozoal genera *Ophryoscolex, Dasytricha* and *Enoploplastron,* fungal family Mucoraceae and genus *Mucor* were found to differ significantly among the four treatment groups. Moreover, Spearman correlation analysis revealed that the ciliate protozoa and fungi were negatively correlated with each other. However, further studies are needed to determine Se affect on the rumen eukaryotic population (protozoa, fungi) associated with rumen prokaryotic community (bacteria, archaea) and the extent of this association.

## Methods

### Ethics statement

Our animal experiment was approved by the Institutional Animal Care and Use Committee of the Yulin University (Ylin, China) under permit number YLU2021-2 and was performed in the Laboratory Animal Center of Cashmere goat research institution located in the suburb of Yuyang District, Yulin, China. All experiments were performed in accordance with the university’s guidelines and regulations for animal research (file no: YLU2021-2), and all methods are reported in the present study in accordance with ARRIVE guidelines (https://arriveguidelines.org).

### Animal, diets and experimental design

Thirty-two three months old Shaanbei white cashmere wether goats with similar body [(26.18 ± 2.71) kg] were selected and randomly assigned into 4 treatment groups. The basal diet was formulated according to Chinese Feeding Standard of Meat-Producing Sheep and Goats (NY/T 816-2004) as described previously^11^, which contained 0.016 mg/kg DM Se as control diet (control group, CG). Three treatment group’s animals with different levels SeHLan addition to make the Se contents as follows: low Se group (LSE: 0.3 mg/kg DM), medium Se group (MSE: 0.6 mg/kg DM), and high Se group (HSE: 1.2 mg/kg DM).

### Sample collection, DNA extraction, PCR amplification and sequencing

The feeding experiment period lasted for 70 days including 10 days adaptation and 60 days of formal feeding trial period. All animals were fed at 09:00 a.m. and 17:00 p.m., with free access to water. On the last day of feeding trial, rumen samples from each goat were collected after 2 h morning feeding using an oral stomach tube. The first 50 mL of rumen samples in each sampling were discarded to prevent the potential saliva contamination, and final 20 mL of rumen sample were collected from each goat for further analysis^11^. Each rumen content sample was immediately sealed in a centrifuge tube and frozen in liquid nitrogen and then stored at -80℃ for eukaryotic diversity analysis.

The total genome DNA of all rumen samples were extracted using the CTAB/SDS method and diluted to 1 ng/µL with sterile water. The V4 hypervariable region of the 18S rRNA gene was amplified with the following primers 528F (5′-GCGGTAATTCCAGCTCCAA-3′) and 706R (5′-AATCCRAGAATTTCACCTCT-3′) as previous studies^50,51^. PCR reaction system contained 15 µL Phusion® High-Fidelity PCR Master Mix (New England Biolabs), 0.2 µM each primer and 10 ng DNA, and cycling program started at 98 ℃ (1 min) for the step of denaturation, followed by 30 cycles at 98 ℃ (10 s), 50 ℃ (30 s) and 72 ℃ (30 s) and a final extension at 72 ℃ for 5 min. PCR products were mixed with an equal volume of 1X loading buffer and detected on a 2% agarose gel. PCR products were purified using Qiagen Gel Extraction Kit (Qiagen, Germany) according to the manufacturer's instructions. Sequencing libraries were constructed with NEBNext® Ultra™ II DNA Library Prep Kit (Cat No. E7645) according to the manufacturer's instructions. The Qubit@ 2.0 Fluorometer (Thermo Scientific) and Agilent Bioanalyzer 2100 system were employed to evaluate the quality of the library. Finally, the library was sequenced on an Illumina NovaSeq platform.

### Data analysis

Based on the unique barcodes, the generated 250 bp paired-end reads were assigned to samples and truncated by cutting off the barcodes and primer sequences, and merged using FLASH (V1.2.7, http://ccb.jhu.edu/software/FLASH/)^52^, which was designed to merge paired-end reads when at least some of the reads overlap the read generated from the opposite end of the same DNA fragment, and the splicing sequences were called raw tags. Raw tags were quality filtered under specific filtering conditions to get the high-quality clean tag^53^ in accordance with the QIIME (V1.9.1, http://qiime.org/scripts/split_libraries_fastq.html)^54^ quality controlled process. The tags were compared with the Silva database (https://www.arb-silva.de/)^55^ using UCHIME algorithm (http://www.drive5.com/usearch/manual/uchime_algo.html)^56^ to detect chimera sequences. Removal of the chimera sequences^57^ to finally got the effective Tags. Clustering of sequence analyses into OTUs by UPARSE software (Uparse v7.0.1001, http://drive5.com/uparse/) based on 97% similarity^58^. Using the mothur algorithm and the SILVA database (http://www.arb-silva.de/) to annotate the taxonomic assignment with a 0.80 confidence threshold and to identify the dominant species within each sample^55^. Using MUSCLE 3.8.31 to perform the multiple sequence alignment^59^. Taxonomical data was amalgamated for each group and relative proportions of the eukaryotes at the genus level in each group were correlated by calculated the Spearman correlation coefficient method through R software (Version 2.15.3) with igraph package.

### Statistical analysis

In this study, alpha diversity indices (Observed_otus, Chao1, Shannon, Simpson, ACE, Good’s coverage and PD_whole_tree) were calculated with QIIME (Version 1.9.1) and displayed in R software (Version 2.15.3) for analyze the diversity, richness and uniformity. Principal coordinate analysis (PCoA) was performed using ade4 package and ggplot2 package in R software (Version 2.15.3) to obtain principal coordinates and visualize differences of samples in complex multi-dimensional data based on Bray–Curtis dissimilarity matrices. A heatmap of the top 35 most abundant eukaryotic genera was constructed using R software (Version 2.15.3) with heatmap package. Comparison of the alpha-diversity indices by one-way ANOVA (IBM SPSS Statistics 20) using a completely randomized design. The comparison for the eukaryotes relative abundances among the four treatments using IBM SPSS Statistics 20 by the Kruskal–Wallis rank sum test. Significant difference value was set at *P* < 0.05.

### Ethics declarations

The authors confirm that this study was performed in accordance with the Guidelines of Good Experimental Practices adopted by the Institute of Shaanxi Provincial Engineering and Technology Research Center of Cashmere Goats, Yulin University, Yulin, China. All experimental protocols involving animals were approved by the Animal Care Committee of Yulin University (Yulin, China) and were under the university’s guidelines for animal research (file no: YLU2021-2).

### Supplementary Information

Supplementary Tables.

## Data Availability

The datasets generated for this study can be found in NCBI Sequence Read Archive, with the access number PRJNA846111.
