# Supplementary material for: The effects of Selenohomolanthionine supplementation on the rumen eukaryotic diversity of Shaanbei white cashmere wether goats
Source: Sci Rep. 2023 Aug 12;13:13134. doi: 10.1038/s41598-023-39953-2 (PMC10423290; doi:10.1038/s41598-023-39953-2)
Supplement: Supplementary file 1 — Supplementary Tables. [file 41598_2023_39953_MOESM1_ESM.doc]

The effects of Selenohomolanthionine supplementation on the rumen Eukaryotic Diversity of Shaanbei white cashmere wether goats

Longping Li ^1^*,Lei Qu^1^ and Tuo Li ^1,2^

^1^Shaanxi Provincial Engineering and Technology Research Center of Cashmere Goats, Yulin University, Yulin 719000, China

^2^College of Life Sciences, Yulin University, Yulin 719000, China

*****Correspondence author. Longping Li, E-mail address: [llp_315@163.com](mailto:llp_315@163.com)

**Supplementary Tables**

**Table S1**. The relative abundance (%) of rumen eukaryotic communities at phylum level among four SeHLan supplemented groups (average relative abundance > 0.1% for at least one group).

| **Kingdom** | **Phylum** | **Treatments^1^** | | | | **SEM** | ***P*-value** |
| --- | --- | --- | --- | --- | --- | --- | --- |
|  |  | **CG** | **LSE** | **MSE** | **HSE** |  |  |
| Eukaryota | Ciliophora | 88.68 | 97.91 | 96.45 | 86.61 | 2.35 | 0.644 |
|  | unidentified_Eukaryota | 5.67 | 1.08 | 1.72 | 7.95 | 1.33 | 0.561 |
|  | Diatomea | 1.76 | 0.24 | 0.41 | 2.63 | 0.45 | 0.157 |
|  | MAST-1 | 0.83 | 0.13 | 0.06 | 0.59 | 0.17 | 0.313 |
|  | Cercozoa | 0.77 | 0.05 | 0.53 | 0.58 | 0.19 | 0.903 |
|  | Picozoa | 0.25 | 0.016 | 0.018 | 0.396 | 0.084 | 0.194 |
|  | MAST-8 | 0.24 | 0.008 | 0.006 | 0.147 | 0.056 | 0.169 |
|  | Protalveolata | 0.38 | 0.19 | 0.29 | 0.37 | 0.065 | 0.780 |
|  | Apicomplexa | 0.23 | 0.18 | 0.02 | 0.16 | 0.056 | 0.477 |
| Fungi | Ascomycota | 27.89 | 42.29 | 49.55 | 43.33 | 4.48 | 0.448 |
|  | unidentified_Fungi | 24.26 | 7.07 | 16.85 | 7.39 | 3.25 | 0.627 |
|  | Basidiomycota | 13.39^c^ | 38.66^a^ | 18.18^c^ | 24.83^b^ | 3.15 | 0.040 |
|  | Mucoromycota | 31.37^a^ | 8.55^c^ | 12.52^c^ | 20.85^b^ | 3.18 | 0.039 |
|  | LKM15 | 0.05 | 0.25 | 0.025 | 1.24 | 0.28 | 0.462 |
|  | Cryptomycota | 1.85 | 1.09 | 1.19 | 0.91 | 0.26 | 0.761 |
|  | Chytridiomycota | 0.84 | 0.44 | 0.12 | 0.59 | 0.12 | 0.418 |

^1^CG, LSE, MSE, and HSE, treatment groups supplemented with SeHLan at 0, 0.3, 0.6, and 1.2 mg/kg

DM, respectively.

**Table S2.** The relative abundance (%) of rumen eukaryotic communities at family level among four SeHLan supplemented groups (average relative abundance >1% for at least one group).

| **Kingdom** | **Phylum** | **family** | **Treatments^1^** | | | | **SEM** | ***P*-value** |
| --- | --- | --- | --- | --- | --- | --- | --- | --- |
|  |  |  | **CG** | **LSE** | **MSE** | **HSE** |  |  |
| Eukaryota | Ciliophora | unidentified_Litostomatea | 87.47 | 97.72 | 96.07 | 84.99 | 2.54 | 0.693 |
|  | unidentified_Eukaryota | unidentified_Eukaryota | 1.17 | 0.089 | 0.16 | 0.83 | 0.25 | 0.497 |
|  | Diatomea | unidentified_Bacillariophyceae | 1.08 | 0.097 | 0.11 | 1.48 | 0.28 | 0.150 |
|  | unidentified_Eukaryota | unidentified_Gymnodiniphycidae | 1.29 | 0.28 | 0.25 | 1.19 | 0.24 | 0.917 |
|  | unidentified_Eukaryota | unidentified_Chromulinales | 0.11 | 0.02 | 0.04 | 1.02 | 0.18 | 0.385 |
| Fungi | unidentified_Fungi | Neocallimastigaceae | 23.49 | 6.42 | 15.61 | 6.65 | 3.29 | 0.767 |
|  | Ascomycota | Pichiaceae | 1.21 | 10.23 | 14.85 | 13.24 | 3.95 | 0.228 |
|  | Basidiomycota | Physalacriaceae | 0.62 | 11.09 | 0.19 | 0.008 | 2.71 | 0.409 |
|  | Basidiomycota | Malasseziaceae | 3.41 | 15.27 | 5.21 | 7.02 | 2.22 | 0.249 |
|  | Mucoromycota | Mucoraceae | 28.43 ^a^ | 7.09 ^b^ | 9.73 ^b^ | 16.33 ^a^ | 3.12 | 0.049 |
|  | Ascomycota | Aspergillaceae | 8.89 | 8.49 | 5.11 | 10.47 | 1.65 | 0.421 |
|  | Ascomycota | Saccharomycetaceae | 3.58 | 0.79 | 5.19 | 3.33 | 1.29 | 0.644 |
|  | Ascomycota | Apiosporaceae | 0.94 | 0.77 | 4.25 | 0.39 | 0.95 | 0.656 |
|  | Basidiomycota | Mrakiaceae | 4.89 | 4.72 | 2.19 | 8.13 | 1.22 | 0.123 |
|  | Ascomycota | Cladosporiaceae | 5.09 | 3.75 | 1.70 | 2.39 | 0.89 | 0.571 |

^1^CG, LSE, MSE, and HSE, treatment groups supplemented with SeHLan at 0, 0.3, 0.6, and 1.2 mg/kg DM, respectively.

^a,b^ Within rows, means without a common superscript differ (*P* < 0.05).

**Table S3.** The relative abundance (%) of rumen eukaryotic communities at genus level among four SeHLan supplemented groups (average relative abundance >1% for at least one group).

| **Kingdom** | **Phylum** | **Genus** | **Treatments^1^** | | | | **SEM** | ***P*-value** |
| --- | --- | --- | --- | --- | --- | --- | --- | --- |
|  |  |  | **CG** | **LSE** | **MSE** | **HSE** |  |  |
| Eukaryota | Ciliophora | *Entodinium* | 60.09 | 68.82 | 61.63 | 49.78 | 3.67 | 0.344 |
|  | Ciliophora | *Ophryoscolex* | 12.57 ^a^ | 7.62 ^b^ | 15.51 ^a^ | 7.87 ^b^ | 3.16 | 0.022 |
|  | Ciliophora | *Polyplastron* | 6.31 | 9.48 | 9.02 | 18.27 | 2.48 | 0.361 |
|  | Ciliophora | *Dasytricha* | 2.59 | 3.41 | 0.91 | 3.49 | 0.48 | 0.181 |
|  | Ciliophora | *Enoploplastron* | 1.34 ^a^ | 0.04 ^b^ | 0.34 ^b^ | 0.18 ^b^ | 0.26 | 0.048 |
|  | Ciliophora | *Isotricha* | 1.08 | 0.57 | 1.48 | 0.89 | 0.22 | 0.589 |
|  | Ciliophora | *unidentified_Litostomatea* | 0.02 | 0.005 | 0.003 | 0.49 | 0.12 | 0.072 |
| Fungi | Ascomycota | *Pichia* | 1.21 | 10.23 | 14.85 | 13.24 | 3.94 | 0.228 |
|  | Basidiomycota | *Flammulina* | 0.62 | 11.09 | 0.19 | 0.008 | 2.71 | 0.090 |
|  | Basidiomycota | *Malassezia* | 3.41 | 15.27 | 5.21 | 7.02 | 2.22 | 0.628 |
|  | Mucoromycota | *Mucor* | 28.43 ^a^ | 7.07 ^b^ | 9.73 ^b^ | 16.33 ^a^ | 3.12 | 0.049 |
|  | unidentified_Fungi | *unidentified_Neocallimastigaceae* | 16.25 | 3.06 | 8.52 | 3.04 | 2.01 | 0.708 |
|  | Ascomycota | *Aspergillus* | 8.89 | 8.49 | 5.11 | 10.47 | 1.64 | 0.421 |
|  | Ascomycota | *Saccharomyces* | 3.41 | 0.54 | 5.01 | 2.62 | 1.24 | 0.512 |
|  | unidentified_Fungi | *Cyllamyces* | 4.96 | 3.06 | 6.52 | 3.41 | 1.32 | 0.654 |
|  | Ascomycota | *Arthrinium* | 0.94 | 0.77 | 4.25 | 0.39 | 0.95 | 0.469 |
|  | Basidiomycota | *Tausonia* | 4.89 | 4.72 | 2.19 | 8.08 | 1.21 | 0.135 |

^1^CG, LSE, MSE, and HSE, treatment groups supplemented with SeHLan at 0, 0.3, 0.6, and 1.2 mg/kg DM, respectively.

^a,b^ Within rows, means without a common superscript differ (*P* < 0.05).
